# Your Performance Is My Concern: A Perspective-Taking Competition Task Affects ERPs to Opponent’s Outcomes

**DOI:** 10.3389/fnins.2019.01162

**Published:** 2019-10-25

**Authors:** Hao Yu, Weizhi Nan, Guochun Yang, Qi Li, Haiyan Wu, Xun Liu

**Affiliations:** ^1^Key Laboratory of Behavioral Science, Institute of Psychology, Chinese Academy of Sciences, Beijing, China; ^2^Department of Psychology, Center for Brain and Cognitive Sciences, School of Education, Guangzhou University, Guangzhou, China; ^3^Department of Psychology, University of Chinese Academy of Sciences, Beijing, China

**Keywords:** in-group bias, gambling task, feedback related negativity, P300, EEG

## Abstract

Previous research has shown that people have more empathic responses to in-group members and more schadenfreude to out-group members. As a dimension of cognitive empathy, perspective-taking has been considered to be related to the enhancement of empathy. We tried to combine these effects through manipulation of a competitive task with opponents and an in-group partner and investigated the potential effect of in-group bias or the perspective-taking effect on outcome evaluation. We hypothesized that the neural activities would provide evidence of in-group bias. We tested it with a simple gambling observation task and recorded subjects’ electroencephalographic (EEG) signals. Our results showed that the opponent’s loss evoked larger feedback-related negativity (FRN) and smaller P300 activity than the partner’s loss condition, and there was a win vs. loss differential effect in P300 for the opponent only. The principal component analysis (PCA) replicated the loss vs. win P300 effect to opponent’s performance. Moreover, the correlation between the inclusion of the other in the self (IOS) scores and FRN suggests perspective-taking may induce greater monitoring to opponent’s performance, which increases the win vs. loss differentiation brain response to the out-group agent. Our results thus provide evidence for the enhanced attention toward out-group individuals after competition manipulation, as well as the motivation significance account of FRN.

## Introduction

As an important aspect of self-representation in social life, the in-group bias refers to the behavioral pattern of people more favoring in-group members than out-group members, which also has been widely explored from both developmental and evolutionary views (Brewer, 1979; Struch and Schwartz, 1989; Van Bavel et al., 2008; Yang et al., 2014; Oestereich et al., 2019). Using a minimal group paradigm, Tajfel (1970) and Tajfel et al. (1971) found that even random group classification could elicit in-group bias and out-group discrimination in subjects. A large number of social psychology studies reported the in-group bias or intergroup discrimination respectively from different aspects, such as, developmental and evolutionary views (Brewer, 1979; Struch and Schwartz, 1989; Van Bavel et al., 2008; Yang et al., 2014; Oestereich et al., 2019). Such in-group bias or out-group discrimination modulates various people’s social behaviors, including both helpful and harmful behaviors (Cikara et al., 2011, 2014; Dickinson et al., 2018).

Apart from social psychology studies, neuroscientific research has also provided evidence for the effect of group identity on people’s emotions or action tendency. Studies have shown that racial group membership modulates brain activities in a pain empathy task (Xu et al., 2009; Contreras-Huerta et al., 2013; Fabi and Leuthold, 2018; Han, 2018). Montalan et al. (2012) adopted Minimal Group Paradigm (participants were randomly assigned to different groups: e.g., underestimator and overestimator group in the dot estimation task) to investigate the empathy preference, and found higher pain empathy to in-group members and lower empathy to out-group members in imagined pain empathy condition. Such brain activity of empathy has been shown to be able to predict the altruism motivation (Mathur et al., 2010; Xin et al., 2018) or costly helping (Hein et al., 2010; Preis et al., 2013). Moreover, as an interesting intergroup emotion, schadenfreude (Dasborough and Harvey, 2017) has also been found in neuroscience (Steinbeis and Singer, 2014; Vollberg and Cikara, 2018). One representative work from Cikara et al. (2011) on soccer fans showed that the win of the favorite team (in-group) and the loss of the rival team (out-group) activate the ventral striatum, which is a reward-related brain region. Taken together, both social psychology and neuroimaging studies indicate that in-group bias has an impact on empathy and intergroup schadenfreude.

Previous studies from Sherif showed competition is a key element in group differentiation (Sherif et al., 1961; Sherif, 1966). Previous group manipulations usually involved competition tasks. For example, studies from Molenberghs (Molenberghs et al., 2012; Morrison et al., 2012) used a team competition task in which participants had to respond as quickly as possible after the “GO” signal. In that case, people do not need to face the opponent directly and interact with them. However, people usually need to anticipate their opponents’ mind in the competition situation. Notably, the empathy level is highly correlated with perspective-taking, which is considered as a key cognitive component of empathy (Davis et al., 1996). Perspective-taking is also a way to reduce intergroup conflicts and improve the intergroup relationship (Cohen and Insko, 2008; Shih et al., 2009; Todd et al., 2011; Böhm et al., 2018). For instance, a study showed that a perspective-taking viewing task improved subjects’ liking and induced more empathic feelings toward another member of the out-group (Shih et al., 2009). Such an intergroup relationship improvement effect from perspective-taking may be attributable to the formation of a “social bond” (Mcdonald et al., 2017). However, how a perspective-taking competition game affects the in-group or out-group’s outcome processing has not been investigated. Therefore, the present study will investigate the outcome evaluation by group membership and perspective-taking competition game manipulation.

Works in the domain of outcome evaluation have identified two key related ERP components: the feedback-related negativity (FRN) and the P300 (Osinsky et al., 2016). FRN is a fronto-central negative deflection that is larger following the presentation of negative feedback (Miltner et al., 1997; Holroyd and Coles, 2002; Hajcak et al., 2005). However, other recent studies have suggested that the FRN can be conceptualized as a positive deflection that is more positive following a reward compared with non-reward outcomes, particularly by principal component analysis (PCA) ERP studies (Holroyd et al., 2008; Foti et al., 2011). FRN is thought to be associated with reward prediction errors in reinforcement learning theory (Holroyd and Coles, 2002; Yeung and Sanfey, 2004), which was challenged by a study showing that FRN reflects the salience errors (Talmi et al., 2013) or expectation (Cao et al., 2015). Further, the P300 component, which is traditionally considered as reflecting the attention process or context-updating (Zhao et al., 2017), has also been suggested to be associated with the motivational significance of reward (Wu and Zhou, 2009) or the valence of the outcome (Hajcak et al., 2005).

Interestingly, studies also showed a “mirror” performance monitoring system in which observing another’s gain or loss also evokes similar FRN, which is called observational FRN (oFRN), as it applies in observation situations (Kang et al., 2010; Wang Y. et al., 2014). Fukushima and Hiraki (2009) found that significant oFRN was elicited only when humans were in observation, not computer players. Further, researchers have investigated the effect of an interpersonal relationship through the ERP correlating to outcome evaluation (Itagaki and Katayama, 2008; Leng and Zhou, 2010; Marco-Pallares et al., 2010). For example, Leng and Zhou explored the different neural responses to friends and strangers when the observer was engaged in the same gambling game and failed to find a differentiation of FRN responses between friends and strangers observations. In another study, they found FRN and P300 responses to win and loss feedbacks similarly increased (Zhou et al., 2010). In summary, FRN and P300 are considered as neural markers for empathy toward an outcome evaluation (Miltner et al., 2003; Fukushima and Hiraki, 2009). However, as far as we know, there has been no study combining group membership and perspective-taking manipulation to examine these effects on outcome empathy.

In the present study, we manipulated a temporary group identity in a competition context and utilized an interactive football game to increase the perspective-taking toward the out-group members. Then we examined the possible differential effect on the partner’s and opponent’s win or loss in a benefit-independent context. It sounds that the in-group member’s outcome evoked a larger “empathy” effect due to in-group favoritism. However, as Galinsky et al. (2008) wrote, “*understanding one’s opponent is valuable for success in competitive interactions*” and “*get inside the head of your opponent*” is crucial for social interaction. Following this view, we also expect enhanced attention regarding an out-group member’s outcome. Therefore, we asked an open question regarding which effect (in-group favoritism or perspective-taking) is more prominent in a simple “gambling observing” task. We aimed to examine two possible effects: the in-group empathy bias effect (e.g., more concern about a partner’s outcome or an opponent’s loss per the schadenfreude effect) and the competition induced attention on opponent effect (e.g., more concern to an opponent’s outcome for the interaction in a perspective-taking competition game).

## Materials and Methods

### Participants

Nineteen right-handed man college students with normal vision (age: 22.90 ± 0.93) from Beijing participated in this study. All participants were recruited through advertisement, with no history of neurological or psychiatric illness and no drug intake. To control the task familiarity, all participants reported have the experience of watching football matches. All procedures were approved by the institutional review boards (IRB) of the Institute of Psychology, Chinese Academy of Sciences. All participants signed the informed consent before the experiment and were paid after the experiment.

### Procedure

The interpersonal reactivity index (IRI) and the inclusion of others in the self (IOS) scale data were collected before the experimental procedure. Before the formal experimental procedure, participants were told that they would join this experiment with other three participants (actually experimenters) at the same time. To make the real participants believe this, we called them and emphasized the experiment time to make sure that everybody arrived on time in case of meeting the other experimenters in the building. Before the formal procedure, all participants were asked to take a look at the other experiment rooms, with or without participants there. Moreover, we asked them to wait for 1–2 min before the first stage if one player was late.

**FIGURE 1:**
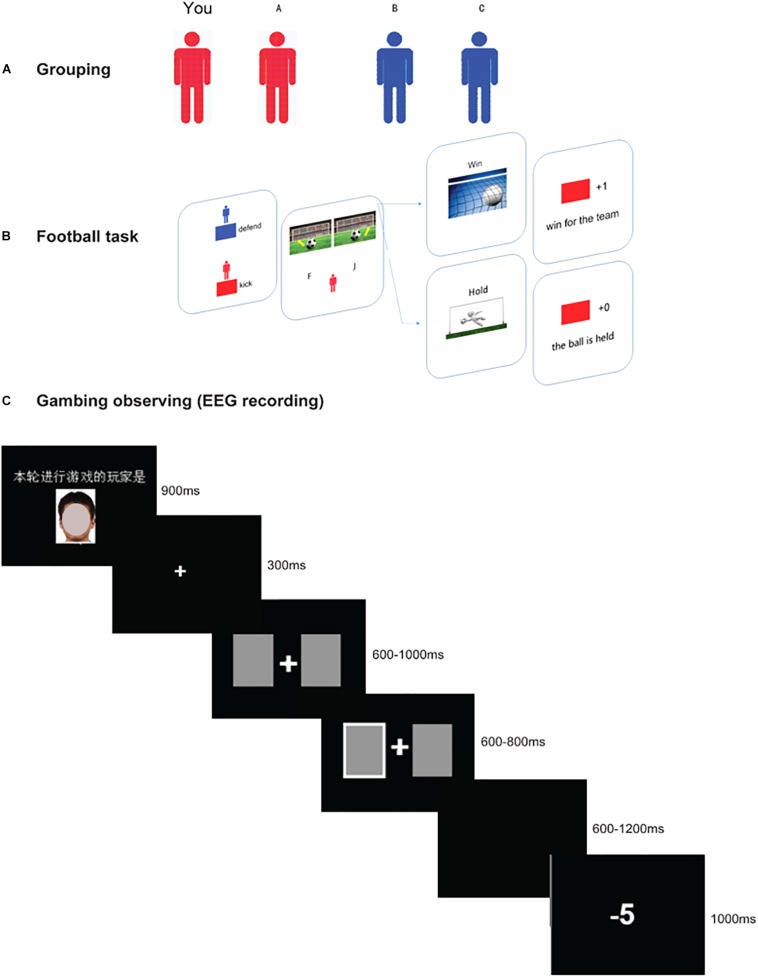
Schematic diagram of the three stages in the present experiment. Panel **(A)** shows the grouping phase, in which participants were randomly arranged in two subgroups (blue team or red team). During this stage, they need to recognize their partner and opponent correctly. Panel **(B)** shows the perspective-taking interactive game. First, the two face a stimulus indicating which two players’ turns it is in this trial. The kicking player, who is presented with a ball in front of a goal, has the option to kick the ball to the left or right. At the same time, the defending player (goalkeeper), also needs to select a side by pressing a button (“F” for the left, “J” for the right). If the goalkeeper saves in the same direction as the kicker’s direction, the defending team wins; otherwise, the kicking team wins. Thus, in this game, the participants need to enter the opponent’s head and choose the opposite direction. The example shows a “win” outcome for the red team. Panel **(C)** shows a two-player gambling task (partner vs. participant) with the time-course of visual stimuli. First, there is a face stimulus (which has been masked to protect the privacy of the participant) indicating which player’s turn it is, followed by two cards (600–1000 ms), and the player is asked to choose either of the cards. The chosen card is indicated (600–800 ms), and a win/loss outcome (1000 ms) is presented after a jitter (600–1200 ms). The example shows a “loss” outcome. In this stage, the players who are not selected to play (e.g., the participant) are asked to observe the other’s performance.

The formal procedure consisted of three stages, as is illustrated in Figure 1.

#### Gambling Observation Stage

Participants were instructed that two players (the partner and one opponent) were randomly selected to participate in the gambling task, and the remaining two players needed to observe their performance. All four players practiced the gambling task and understood that the gain or loss was independent (i.e., win or loss for yourself). For observers, they are asked to answer one question to ensure their involvement in the task. The question is about who would win more money in the gambling task. The one with the correct answer will be awarded 10 Yuan. For each trial of the observation task, the face of the gambling player was presented for 900 ms, implying the one performing the gambling task in the current trial. Then the gambling task started with a fixation for 300 ms, then two cards were presented (two white rectangles with a 2.5° × 2.5° visual angle), and the player was asked to make a selection by pressing the “F” or “J” key. Feedback was presented for 1000 ms at the end of the trial (see Figure 1C). The probability of win/loss was equal across the partner and opponent to rule out potential confounding influences on the differential win–loss probability. There were 200 trials (50 trials for each condition: partner-win, partner-loss, opponent-win, and opponent-loss) in total, with a short break for every 40 trials in the task. The whole gambling observation task lasted around 30 min.

In the formal procedure, all instructions were presented through PowerPoint software. All aforementioned procedures were conducted by E-Prime software (Version 2.0, Psychology Software Tools, Inc.). After completing all formal procedures, participants were asked to rate the performance on a seven-point scale of all players in the tasks (foot task and gambling task). After receiving payment, the participants were also asked to report their involvement or seriousness in the observation task on an assumed 7 point scale.

#### Post-experiment Rating

After the experiment, participants were instructed to recall some experimental details and provide ratings about their feelings of happiness when the other’s win or loss outcome occurred. All participants made a correct recall of their performance in the experiment, and they were paid 10 Yuan by Alipay for their completion of this rating task.

### Electroencephalographic Recording and Preprocessing

During the task, the participant sat approximately 80 cm from a computer screen comfortably in an electrically shielded room. We recorded the electroencephalographic (EEG) data using a 64-channel Neuroscan system (Neuroscan Inc., Herndon, VA, United States) in the gambling observation sessions. Raw EEG data were sampled at 500 Hz/channel, referenced to the left mastoid on-line, with impedance lower than 5 kΩ. Vertical electrooculograms (VEOG) were recorded supra- and infra-orbitally at the left eye. Horizontal EOGs (HEOG) were recorded by electrodes at the left and right orbital rims. The online continuous data were digitized with a bandpass of 0.05–100 Hz.

Electroencephalographics were re-referenced to the average of the left and right mastoids and filtered with a low pass of 20 Hz (24 dB/oct) off-line (Hajcak et al., 2006). Epochs were feedback-locked, from 100 ms before the feedback onset to 500 ms after the feedback onset. Ocular artifacts were removed from the EEGs using a regression procedure implemented with Neuroscan software (Scan 4.5). Trials exceeding the threshold of ±80 μV were excluded from further analysis. As a result, 13.4% of the epochs were rejected across participants. Trials of four conditions (partner-win, partner-loss, opponent-win, and opponent-loss) were averaged, respectively, and a −100 to 0 ms baseline was used to perform a baseline correction.

### Average ERP Analysis

**FIGURE 2:**
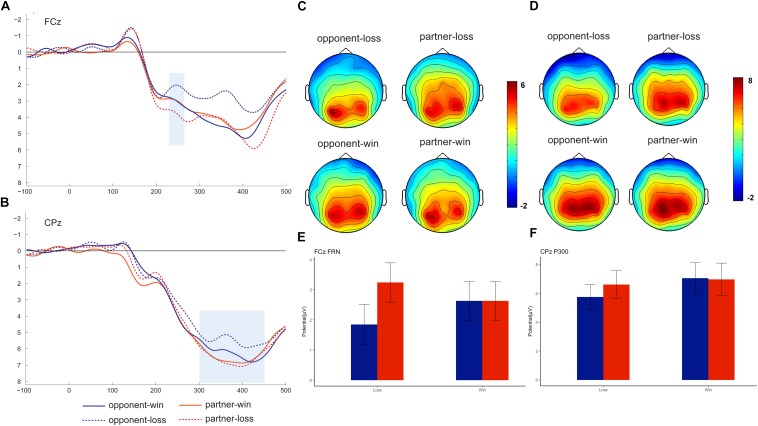
Grand-averaged event-related brain potentials (ERPs). ERPs time-locked to the outcome stimuli at FCz (**A**, mean: **E**) and CPz (**B**, mean: **F**), with the topographical maps for FRN **(C)** and P300 **(D)**.

Previous literature identified FRN by creating a difference wave between win and loss trials (Dunning and Hajcak, 2007; Leng and Zhou, 2010) or from the grand-averaged waveform (Luo et al., 2014). In our study, we are interested in the group effect on ERPs in both the win and the loss conditions. Therefore, we directly measured the FRN and P300 in the grand-averaged waveforms rather than the difference wave. The grand-averaged ERPs at FCz and CPz and the corresponding topography map are presented in Figure 2. Based on both the previous literature (Gu et al., 2011) and visual inspection of the topography map, the FRN was detected at nine fronto-central electrodes (FP1, FPz, FP2, F1, Fz, F2, FC1, FCz, and FC2) and P300 was detected at nine centro-parietal electrodes (C1, Cz, C2, P1, Pz, P2, CP1, CPz, and CP2). Because we are not interested in the electrode effect in the current study, we pooled the nine electrodes and computed the averaged FRN and P300 amplitudes. The FRN amplitude was measured for each participant as the mean amplitude within the 230–280 ms window, while the P300 was identified as the mean amplitude within the 300–450 ms window. Because we are not interested in the electrode effect in the current study, the averaged FRN and P300 amplitudes were entered into a 2 (feedback valence: win and loss) × 2 (agent: partner and opponent) repeated-measures analysis of variance (ANOVA).

### Temporospatial PCA

It is possible that components overlapping in our grand-averaged waveforms, especially for FRN (see Figure 2) and the PCA, is a useful tool for the decomposition of ERPs (Foti et al., 2011). Therefore, we also applied temporo-spatial PCA to more clearly identify the FRN and P300 components. PCA Toolkit (EP Toolkit, version 2.23) and MATLAB 7.8 (MathWorks, Natick, MA, United States) were employed to conduct the PCA in this study (Dien, 2010). We first imported the averaged ERPs from the four conditions for each subject to the toolbox. After checking the data for all conditions, a two-step PCA procedure was performed as in the previous study (Zhang et al., 2013), that is, a temporal PCA was performed on all-time points from each participant’s average ERPs, with promax rotation. After capturing the variances in the time domain, a spatial PCA was conducted for each of the resultant temporal factors using all of the recording electrodes with an infomax rotation. Finally, three temporal factors × three spatial factors were extracted from our ERP data based on the screen plot, yielding nine temporospatial factor combinations. For our specific interest in FRN and P300, we identified these two components and extracted the amplitudes, which were also put into a 2 (feedback valence: win and loss) × 2 (agent: partner and opponent) repeated-measures ANOVA. All ANOVAs in the current study were with Tukey *post hoc* testing at a significance level of 0.05. The significant *p*-value was set as 0.05, and the effect size was calculated using partial eta squared.

## Results

### Behavioral and Psychological Data

The mean IRI score was 69 ± 8.30 (SD) and the IOS score was 4.47 ± 1.26. There was no significant difference between the performance rating for the partner (3.53 ± 1.02) and opponent (3.63 ± 0.76) in the football task, *t* (18) = −0.35, *p* = 0.73. The performance rating of the gambling task between the partner (3.73 ± 0.93) and opponent (3.42 ± 0.69) was also not significantly different, *t* (18) = 1.37, *p* = 0.19.

As an important validation index of the grouping manipulation, the self-reported involvement or seriousness score was 4.05 ± 0.91, showing a relatively high involvement in the observation task. Regarding the football task, 47.3% of the participants (9 participants) were defeated in this game with their teammate and 52.7% won (10 participants), as the random manipulation regulated. Because the winning was equal for the “partner” and “opponent” in the gambling task, 42.1% of the participants (8 participants) chose the opponent and 57.9% chose the partner in the “who wins more” question after the observation task, and the difference of choice probability was not significant. The 2 (outcome valence) × 2 (agent: partner vs. opponent) ANOVA on the happiness rating showed a significant outcome × agent interaction effect, *F* (1, 18) = 41.53, *p* = 0.001, η*_*p*_*^2^ = 0.698). The *post hoc* analysis showed an in-group bias and a schadenfreude effect that the happiness rating was significantly higher when the partner win (5.21 ± 0.15) than when he loses (2.68 ± 0.27), as well as when the opponent loses (4.74 ± 0.30) rather than opponent win (3 ± 0.24), *p*s = 0.01.

### Grand-Averaged ERP Results

#### FRN Component

The repeated-measures 2 (outcome valence) × 2 (agent: partner vs. opponent) ANOVA showed a significant interaction outcome × agent effect, *F* (1, 18) = 12.73, *p* = 0.02, η*_*p*_*^2^ = 0.415). Further analysis indicated that the FRN was more negative-going following the opponent’s losses (1.85 ± 0.66 μV) than following the partner’s losses (3.24 ± 0.65 μV), *F* (1, 18) = 8.01, *p* = 0.01. We did not find significant FRN results between opponent’s wins (2.63 ± 0.42 μV) and partner’s wins (2.63 ± 0.42 μV).

#### P300 Component

The repeated-measures 2 (outcome valence) × 2 (agent: partner vs. opponent) ANOVA on the P3 amplitude also showed a significant outcome × agent interaction effect, *F* (1, 18) = 5.51, *p* = 0.031, η*_*p*_*^2^ = 0.245). Further analysis indicated a smaller P300 for the opponent’s losses (5.25 ± 0.81 μV) than for the partner’s losses (6.24 ± 0.89 μV), *F* (1, 18) = 4.86, *p* = 0.03. The *post hoc* analysis also indicated a significant difference for the opponent’s win (6.53 ± 1.01 μV) versus opponent’s loss, *F* (1, 18) = 4.89, *p* = 0.041, while such a win vs. loss difference was not significant for the partner, *p* = 0.67.

### PCA ERP Results

**FIGURE 3:**
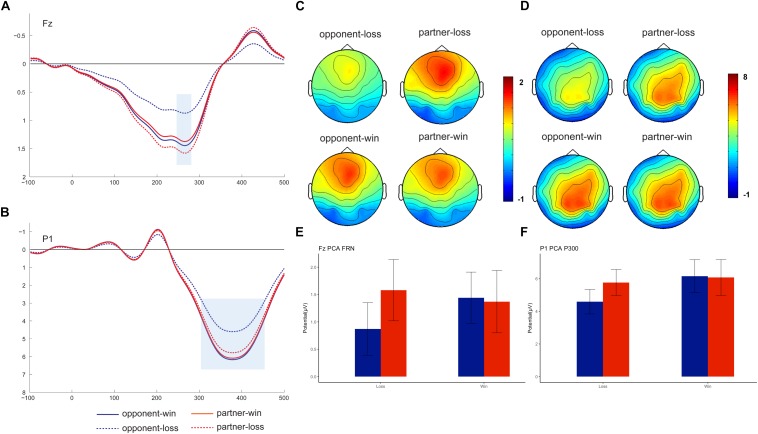
Grand-averaged PCA-FRN and PCA-P300. PCA-FRN components on the peak channel of Fz (**A**, mean: **E**) and PCA-P300 component on the peak channel of P1 (**B**, mean: **F**), with a topographical map showing the fronto-centro FRN **(C)** and centro-parietal P300 **(D)**.

Nine-factor combinations consisted of three temporal factors and three spatial factors (see Supplementary Table 1). We identified the FRN (peaked at Fz on 266 ms) and P300 (peaked at P1 on 378 ms) based on the visual inspection of the factor combinations and the previous PCA results (Foti et al., 2011; Zhang et al., 2013) (see Figure 3). These two PCA components were statistically analyzed as the mean amplitudes within different time windows (250–300 ms for PCA-FRN, 300–450 ms for PCA-P300) at their peak channels (i.e., Fz and P1). Thereafter, the mean values of the amplitudes were separately subjected to repeated-measures 2 (outcome valence) × 2 (agent: partner vs. opponent) ANOVAs.

As Figure 3 shows, we found a PCA-FRN component that was prominent in the fronto-central brain area. However, the outcome × agent ANOVA on the PCA-FRN amplitude failed to find a significant main effect or interaction effect, *F*s < 2.50, *ps* > 0.13.

For the PCA-P300 component, the outcome × agent ANOVA showed a nearly significant main effect of the outcome, *F* (1, 18) = 3.39, *p* = 0.08, η*_*p*_*^2^ = 0.158. Moreover, there was a significant outcome × agent interaction effect [*F* (1, 18) = 7.34, *p* = 0.014, η*_*p*_*^2^ = 0.290], confirming the smaller PCA-P300 for the opponent’s losses (4.59 ± 0.75 μV) was relative to following the partner’s losses (5.77 ± 0.80 μV), *F* (1, 18) = 6.85, *p* = 0.017. Additionally, there was only a significant PCA-P300 win vs. loss difference for the opponent, *F* (1, 18) = 6.72, *p* = 0.018.

To confirm the component identification, the correlations between the PCA components and the grand-average components were computed. The correlation analysis showed a significant correlation between FRN and PCA-FRN, with a Pearson correlation of 0.826, *p* = 0.01. Similarly, the P300 amplitude and PCA-P300 amplitude are also very significant (Pearson correlation *r* = 0.954), *p* = 0.01, which confirmed our PCA component analysis.

### Correlation Between Questionnaire Data and ERP Results

**FIGURE 4:**
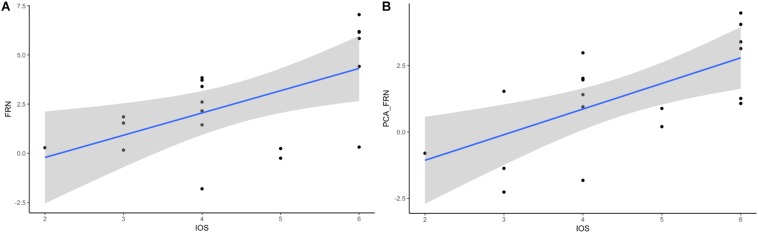
Relationship of FRN-IOS and PCA_FRN-IOS. **(A)** FRN-IOS correlation. **(B)** PCA_FRN-IOS correlation.

A previous study showed the association between the IOS scale and the oFRN component (Kang et al., 2010) and the relationship between oMFN and IRI scores (Fukushima and Hiraki, 2009). Therefore, we performed a correlation between the questionnaire (IRI and IOS) scores and the ERP amplitudes (i.e., FRN, P300, PCA-FRN, and PCA-P300). Interestingly, we also found a significant correlation between the IOS score and FRN component (see Figure 4A, *r* = 0.565), *p* = 0.012, and the correlation between the IOS score and the PCA-FRN component (see Figure 4B, *r* = 0.640) was also significant, *p* = 0.01. These correlation results indicate that participants’ perspective taking increases as the self-other overlap increases. However, no correlation was found for the IRI scores or P300, and there was also no significant correlation between the win/loss or performance rating and the ERP components.

## Discussion

We examined the group membership effects on the outcome evaluation. The observation of the others’ win or loss can be used as a window to investigate the reaction to the in-group or out-group members’ performance. The behavioral rating on the football task and gambling showed no “in-group favoritism.” For example, the participants showed no partner vs. opponent performance difference in either task. Considering that the probability of win/loss was made random in the football task and equal in the gambling task, equal performance for the players is reasonable, and it ruled out potential confounding influences of the differential win/loss probability. However, the happiness rating after the experiment showed an in-group bias and schadenfreude effect that the happiness rating was significantly higher when the partner won or the opponent lost. Such an effect confirms the group membership manipulation was successful. Nevertheless, the rating could also be attributed to the expected effect or the “participant demand characteristics” (Nichols and Maner, 2008), that participants may think the experimenter expected them to show an in-group preference in the rating.

For the EEG results, our results first showed the same increase pattern of FRN-P300 components in outcome processing while observing the performances of the non-self agents (the component becomes more negative at opponent loss condition). These two components were further confirmed by the PCA analysis which is consistent with the findings of the previous study (Zhou et al., 2010). Moreover, the ERP data suggested that the amplitude of the FRN and P300 reflected the interaction between the outcome and agent. Interestingly, a prominent win vs. loss differentiation FRN effect was observed on the opponent, i.e., the loss feedback evoked a more negative deflection than win feedback. The win vs. loss differentiation effect of the FRN was consistent with many previous studies’ (Miltner et al., 1997; Holroyd and Coles, 2002; Hajcak et al., 2005; Wei et al., 2015; Zheng et al., 2017), but we have not found this win vs. loss differentiation effect on partner’s outcomes. Our FRN results seem to reflect the influences of social factors in this early stage of outcome evaluation, which was consistent with the previous study (Qi et al., 2018). However, further PCA-FRN failed to replicate this effect for non-significant interaction effects. One possibility is that the PCA factor is not large enough to reach a significant differentiation effect, even it shows a similar opponent’s win vs. opponent’s loss difference pattern (Figure 3A) as the ERP results (Figure 2A). Another possibility is that the early FRN is not sensitive to social relationships, as it may be entangled with the P300 effect, which is consistent with a previous study showing an interpersonal effect on P300 but not on FRN (Leng and Zhou, 2010).

Likewise, the P300 component showed a similar win vs. loss differentiation effect on the opponent’s feedback, but not for the partners. Specifically, the opponent’s feedback P300 was more positive for the win trials than for the loss trials. Although whether the feedback P300 is sensitive to the outcome valence is still controversial (Yeung and Sanfey, 2004; Gu et al., 2011), we could infer that there is more “empathy” or concern about the opponent’s feedbacks, as it would be consistent with other studies showing a win vs. loss P300 effect on the self’s and other’s feedback (Wu and Zhou, 2009; Leng and Zhou, 2010). The PCA-P300 results confirmed such greater concern about the opponent’s win and loss as the differentiation between win vs. loss. Thereafter, we also observed a partner vs. opponent differential effect in the loss context, showing a smaller P300 and PCA-P300 for the opponent’s losses than for the partner’s losses. Such an effect, we believe, is attributed to the pronounced opponent loss P300 effect. Unlike Leng and Zhou’s (2010) study which showed that P300 was independently modulated by an interpersonal relationship and outcome valence, our results showed the modulation effect of the interaction. The comparison of our ERP results with previous studies in an observation situation (Fukushima and Hiraki, 2006, 2009; Leng and Zhou, 2010; Wang Y. et al., 2014) found FRN and P300 in negative feedback trials became more negative compared to positive feedback trials, for the opponent only. Thus, we can conclude that the participants showed more empathy (perspective-taking) or concern for the opponent’s outcome, which manifested a win vs. loss ERP differentiation effect.

As mentioned, a growing number of studies have suggested that outcome evaluation/empathy ERPs are influenced by the relationship between the agent and the observer (Itagaki and Katayama, 2008; Marco-Pallares et al., 2010; Leng and Zhou, 2010; Qi et al., 2018). In general, an experiment designed with close others or others with a higher self-resemblance will cause participants’ larger FRN or P3 (Fukushima and Hiraki, 2009; Leng and Zhou, 2010). Together with previous evidence showing more empathy to in-group members (Xu et al., 2009; Contreras-Huerta et al., 2013), the ERP results showed no win vs. loss differentiation effect on the in-group partner, which seemed to be particularly surprising at first sight. The gender of the participants and the group manipulation may account for the partner’s indifferent attitude. Because winning or losing did not change the participants’ bonuses, the in-group control may not be as effective as out-group control. As several studies suggested, man subjects have a lesser empathetic response than women (Fukushima and Hiraki, 2006; Tousignant et al., 2017). More importantly, recent work showed that women’s ERP response of outcome processing was influenced by a short-term induced affective preference, but not that of men (Wang Y. et al., 2014). Furthermore, previous brain imaging studies showing an in-group bias are mainly based on racial or relative long-term social identity (Xu et al., 2009; Contreras-Huerta et al., 2013; Fabi and Leuthold, 2018; Han, 2018). According to male-warrior hypothesis that males respond much stronger sense of competition and are more aggressive in social context (Björkqvist et al., 1994; White and Kowalski, 1994; Van Vugt et al., 2007; Van Vugt, 2009), the man-only participant population may lead to differentiation to opponent’s results. Considering that the group identity manipulation in the current study was short-term and temporary, it is interpretable that the men would show very less concern about their partner’s performance when the outcome was not related to their own self-interest, but about the opponent’s as the potential competition.

However, the brain potential responses showed win vs. loss differential effects on the opponent, which seems like an empathy effect. We noted that one previous ERP study showed both empathy and schadenfreude effects (Itagaki and Katayama, 2008). In Itagaki and Katayama’s study, the other’s loss elicited FRN (loss-win) under cooperative conditions (i.e., empathy), while the observation of the gain of player A also elicited an FRN in player B under antagonistic conditions (i.e., schadenfreude). Unlike their research, the observation task in our study was neither cooperative nor antagonistic, for the agent’s outcome was irrelevant to self-benefit. Therefore, the observer was in a neutral position while viewing the partner’s and opponent’s performances, which was confirmed by the performance rating for the two agents. Thus, we did not find a schadenfreude effect that the opponent’s win evoked a more negative FRN.

By contrast, we found an empathy-like pattern on the opponent’s outcomes, that the opponent’s loss evoked a more negative FRN and P300. We inferred that the perspective-taking strategic game leads to such an effect. Since the participants have social interaction with the opponent but not partners in the game understanding the opponent’s mind is critical for winning the game. Previous work has shown the effect of perspective-taking on decreasing racial bias (Todd et al., 2011; Bimper, 2015; Todd and Simpson, 2016; Müller and Scherr, 2017) and stereotypic bias (Galinsky and Moskowitz, 2000; Wang et al., 2018; Mishra et al., 2019) and increasing the willingness for intergroup contact (Wang C.S. et al., 2014). More importantly, the effect of perspective-taking on improvement in intergroup attitudes was mediated by empathy (Vescio et al., 2003). Combined with Lamm’s et al. (2014) study showing that perspective-taking increases empathy, we inferred that the interaction in the competition task reinforced an empathic-like ERP pattern toward the opponents. When participants take the perspective of the opponent, there was a greater overlap between the mental representations of the self and the agent (Davis et al., 1996). The higher self-other overlap results in empathy toward the opponent, which is also confirmed by the correlation between the IOS scores and the FRN component. Therefore, our results provided a shred of evidence for the self-other overlap framework that proposes perspective-taking induces a self-other overlap and further increases social cooperation and ultimately formation of social bonds (Galinsky et al., 2005).

Another interpretation of the win vs. loss differentiation to opponents’ performance is that the motivation significance is relatively higher for participants. That is, existing studies have shown FRN was modulated by motivation level. For instance, the FRN was smaller when they observe the others’ performance than FRN in joint action (Loehr and Vesper, 2015; Michel et al., 2018). In our study, to some extent, the competitive task has reinforced the motivation to observe the opponents’ outcomes due to the interactive game against opponents. Additionally, all the participants in the current study are men, who have higher competitiveness and win orientation in the sports domain (Gill, 1988; Gill et al., 1996), this competitive attitude may increase the motivation to monitor the opponent’s performance. A recent study has also confirmed that people showed larger FRN in competitive instruction than in cooperative instruction (Cui et al., 2016).

We admit that the small sample size and the lack of a control group may limit generalization of the conclusion (Christley, 2010)^1^. It would also be very interesting to determine the woman’s empathetic response under grouping and perspective-taking manipulation. In the present study, our subjects only included man subjects for several reasons. First, existing studies showed a gender difference in empathy (e.g., women showed more empathetic responses to others) (Han et al., 2008; Lim et al., 2018; Yue et al., 2018) and schadenfreude (e.g., men exhibit more schadenfreude toward others) (Singer et al., 2006). Furthermore, the hormones oxytocin (Shamay-Tsoory et al., 2009; Tetsu et al., 2015) and testosterone (Christian and Shariff, 2017) are associated with schadenfreude or empathy, which also modulate the mentalizing network (Wu et al., 2018). Therefore, we only examined the two effects in man subjects to exclude confounding gender or hormonal factors and investigated the FRN-P300 effect of empathy. Future work that would extend these issues to other situations can provide further evidence about the interaction effect between perspective-taking and group identity on empathy. However, the higher perspective-taking opponent and temporary in-group partner manipulation in the current study only exhibited an empathy pattern to the opponent’s outcome. Although the effect of PCA-FRN is not significant, such an effect of the more negative FRN and P300 in response to an opponent’s loss versus an opponent’s win was observed in the ERP results and PCA-P300. We also look forward to combining perspective-taking, EEG, source localization and connectivity in the future study (Liu et al., 2019) to further investigate this research question.

In summary, our results find neither an in-group bias in empathy nor an intergroup schadenfreude pattern as predicted by the in-group-favoritism hypothesis. Instead, the results of empathy toward the opponent’s outcome are consistent with the perspective-taking and self-other overlap hypotheses. Our results also provide a positive view of improving intergroup relationships and forming social bonds by perspective-taking or social interaction.

## Data Availability Statement

The datasets generated for this study are available on request to the corresponding author.

## Ethics Statement

The studies involving human participants were reviewed and approved by the Institutional Review Boards (IRB) of the Institute of Psychology, Chinese Academy of Sciences. The patients/participants provided their written informed consent to participate in this study.

## Author Contributions

HY: whole experiment execution manuscript drafting and part of the statistical analysis. WN and GY: part of the statistical analysis and refine manuscript. QL: refine manuscript and discussion result. HW and XL: supervise whole experiment design, discussion result, and suggestions for organize manuscript draft.

## Conflict of Interest

The authors declare that the research was conducted in the absence of any commercial or financial relationships that could be construed as a potential conflict of interest.

## Footnotes

1 Using G^∗^Power (Faul et al., 2007), we calculated that with a sample size of 19, with the current 2 × 2 design, we had a power of β = 0.80 to detect a very large effect, namely effects that are larger than *p*η^2^ = 0.34/f.72. For a large sized effect (*p*η^2^ = 0.14/f.40) the current study had a power of β = 0.35, and for a medium sized effect (*p*η^2^ = 0.06/f.25) a power of β = 0.17. Hence we have to admit that the small sample size severely limited the generalization of the conclusions.
