# Supplementary material for: Your Performance Is My Concern: A Perspective-Taking Competition Task Affects ERPs to Opponent’s Outcomes
Source: Front Neurosci. 2019 Oct 25;13:1162. doi: 10.3389/fnins.2019.01162 (PMC6829177; doi:10.3389/fnins.2019.01162)
Supplement: Supplementary file 1 [file Table_1.doc]

Table 1

Summary of the PCA factors

| factor  number | PCA  factor ^a^ | associated  ERP component | variance  explained (%) | positive or negative | peak  latency (ms) | peak  channel |
| --- | --- | --- | --- | --- | --- | --- |
| 1 | TF1SF1 | SW | 23.12 | + | 532 | F6 |
| 2 | TF1SF2 |  | 11.79 | - | 532 | FPz |
| 3 | TF1SF3 |  | 2.57 | + | 532 | FT8 |
| 4 | TF2SF1 | P3 | 23.88 | + | 378 | P1 |
| 5 | TF2SF2 |  | 7.91 | + | 378 | FPz |
| 6 | TF2SF3 |  | 1.06 | + | 378 | F8 |
| 7 | TF3SF1 | FRN | 4.07 | + | 266 | Fz |
| 8 | TF3SF2 |  | 2.24 | + | 266 | PO6 |
| 9 | TF3SF3 |  | 0.77 | **+** | 266 | F8 |
|  |  |  |  |  |  |  |

^a^ TF = temporal factor, SF = spatial factor, SW = slow wave. For the sake of brevity, the SW component was not presented and discussed in the text.
